# Lethal Pneumonia Cases in Mojiang Miners (2012) and the Mineshaft Could Provide Important Clues to the Origin of SARS-CoV-2

**DOI:** 10.3389/fpubh.2020.581569

**Published:** 2020-10-20

**Authors:** Monali C. Rahalkar, Rahul A. Bahulikar

**Affiliations:** ^1^C2, Bioenergy Group, MACS Agharkar Research Institute, Pune, India; ^2^BAIF Development Research Foundation, Central Research Station, Pune, India

**Keywords:** RaTG13, SARS-CoV-2, pneumonia, mineshaft, Mojiang, origin, COVID-19

## Abstract

With the COVID-19 pandemic reaching its worst heights, people are interested in the origin of SARS-CoV-2. This study started with two important questions: first, were there any similar atypical pneumonia outbreaks, even on a smaller level, reported between SARS in 2004 and COVID-19 in 2019/20 in China. Second, examining the beta-coronavirus most closely related to date with SARS-CoV-2 at the genome sequence level, strain RaTG13 (CoV4991), which was sampled from a horseshoe bat in Yunnan province, we asked where exactly did it come from. It was found that RaTG13/CoV4991 was collected from Tongguan mineshaft in Mojiang, Yunnan, China, in 2013. Surprisingly, the same mineshaft was also associated with a severe pneumonia-like illness in miners in 2012 killing three of the six miners. A Master's thesis (in the Chinese language) was found on the cnki.net website which described in detail the severe illness in miners. The thesis concluded that a SARS-like CoV originating from Chinese horseshoe bats (*Rhinolophus*) was the predicted causative agent. The cases were remotely monitored by a prominent pulmonologist in China. Retrospective analysis of the pneumonia cases shows striking similarities with COVID-19. Bilateral pneumonia, vascular complications like pulmonary thromboembolism, and secondary infections are the main similarities. The treatment regimes were similar to the current treatments for COVID-19. We propose that the Mojiang mineshaft miners' illness could provide important clues to the origin of SARS-CoV-2. These cases should be studied by various academicians, researchers, and medical professionals as many important questions are raised in this context.

## One Line Summary

Lethal pneumonia in Mojiang miners and the mine could provide an important link to the research investigating the origin of SARS-CoV-2.

## Introduction

The global COVID-19 pandemic has now affected more than 26 million people with a death toll of 0.8 million affecting 188 countries and territories. Horseshoe bats (*Rhinolphus* sp.) are considered to be the natural hosts or reservoirs of the Severe Acute Respiratory Syndrome (SARS)-CoV and SARS-CoV-2, the causative agent of the current pandemic COVID-19 (1). The horseshoe bats usually inhabit the Yunnan Province in China's southern sub-tropical zone (2) and Yunnan is also the likely region for the origin of SARS-CoV-2 (3).

### Mojiang Mineshaft Associated With Lethal Pneumonia Cases in Miners (2012)

This study scientifically investigated any reports of atypical pneumonia cases covering the period 2004 to 2019, between the SARS (1) epidemic and COVID-19. We identified a case described in two scientific magazines. The first mention was in an interview with Dr. Zhengli Shi, a principal scientist of WIV, in the Scientific American journal (2). In the interview, Shi talked about a mineshaft in Mojiang where a lethal pneumonia-like disease occurred in six miners in 2012 (2). The discussion outlined that a diverse group of coronaviruses was discovered in the mine following the outbreak. These lethal pneumonia cases were also covered in news, including a more detailed article in Science magazine in 2014 (4). In April 2012, a pneumonia-like illness occurred in six miners who were cleaning bat feces from a copper mineshaft in Mojiang, Yunnan, in 2012, killing three of them (4). The Science article describes that a paramyxovirus, MojV was isolated from a rat sample in the same mine (4). Two further papers reported that no direct relationship between human infection and MojV could be established (4–6). In the Scientific American interview, Dr. Zhengli Shi outlines that fungus was responsible for pneumonia in the miners (2). However, no detailed information was elucidated in literature and the cause of the miners' illness remained a mystery.

### Master's Thesis by **Li Xu** on the “Mojiang Miners Pneumonia” Illness

In 2013, Li Xu published a Master's thesis (7) that described in detail the symptoms suffered by six pneumonia patients. This thesis was found by a twitter user (@TheSeeker268), who emailed us the link after reading our pre-print (8) (on May 20, 2020). The thesis was found on the cnki.net website which is the official website for Master's and Ph.D. thesis in China and therefore considered to be a valid source. The original thesis is in Chinese (7) and we translated it using google translation. Currently, a professional translation of the thesis has been made available online by a research agency (https://bioscienceresource.org/) that is currently examining the Master's thesis (https://www.documentcloud.org/documents/6981198-Analysis-of-Six-Patients-With-Unknown-Viruses.html).

According to the Master's thesis, in April 2012, six miners were given a job of clearing bat waste and bat feces from a copper mineshaft in Tongguan, Mojiang, Yunnan. After working for ~14 days in the case of four miners, and 4–5 days in the case of the last two miners, they started facing breathing problems, cough, and fever which required immediate admission to the Kunming hospital in late April and early May (7). Three of the miners died in the course of ~100 days and three survived (Table 1A). The thesis featured medical reports, radiological images such as CT scans, and detailed information regarding the diagnosis and treatment of the miners (7). (https://www.documentcloud.org/documents/6981198-Analysis-of-Six-Patients-With-Unknown-Viruses.html).

**Table 1A:** Summary of the six pneumonia patients in 2012 [as per (7)].

Number of the patient^*^	**Age**	**Admitted to the hospital on**	**Worked in the mine for**	**Days in the hospital**	**Outcome/date of discharge/death**
1.	63	26.04.2012	14 days	12	**Death** 07.05.2012
2.	42	25.04.2012	14 days	48	**Death** 12.06.2012
3.	45	27.04.2012	14 days	109	**Death** 13.08.2012
4.	46	26.04.2012	14 days	107 (actual days 137)	Improved and discharged on 10.09.2012
5.	30	02.05.2012	5 days	26	Alive, discharged on 28.05.2012
6.	32	26.04.2012	4 days	32	Alive, discharged on 28.05.2012

* *Names not given*.

### Severe Pneumonia and Illness in the Mojiang Miners Related to Horseshoe Bats in the Mojiang Mine (7)

The main clinical symptoms in the six patients from the Mojiang mine were cough and fever, and the main accompanying symptoms were dyspnoea, aching limbs, sputum/bloody sputum, and headache. The details of the course of illness and diagnosis for individual patients are summarized in Supplementary Information A. Radiography showed interstitial pneumonia, ground-glass opacities, and severe acute respiratory distress syndrome (ARDS) in the first four patients who also required a mechanical ventilator (patients 2–4). Some patients (1, 2, and 4) showed clotting complications such as pulmonary thromboembolism or thrombosis and elevated D-dimer values. Dr. Zhong Nanshan, a doctor for respiratory diseases and a national advisor for the SARS and COVID-19 epidemic, had provided remote consultation for patients 3 and 4, the most serious patients. Patients 3 and 4 remained in the hospital for more than 100 days. Four patients (1–4) a very low oxygenation index and classified as ARDS (Berlin criteria, 2012). Dr. Nanshan's diagnosis for patients 3 and 4 were interstitial pneumonia (primarily of viral origin), with a possibility of secondary infection (invasive pulmonary aspergillosis). He requested swab testing and SARS antibody testing (to be carried in WIV). He also asked the hospital staff to confirm with the Kunming Institute of Zoology for the type of bat. The radiological findings were diffuse ground-glass opacities and areas of peripheral consolidation. The thesis concluded that the pneumonia cases were due to viral pneumonia, primarily from SARS-like coronaviruses originating from horseshoe bats. The percentage of lymphocytes, T, B, and NK cells decreased significantly after the admission of the patients, which indicated that the immune system of the patients was seriously damaged by a viral infection. Later, after the consultation of Dr. Zhong Nanshan, (~after June 19, 2012), blood samples were sent to WIV for antibody testing. A chapter in a Ph.D. thesis by Canping Huang (supervised by Dr. George Gao, present Director China CDCP) also highlights these cases (9) (a translation of Chapter 3 is provided as Supplementary Material). According to the translation of the Ph.D. thesis (Lines 283–285, page 9), the “blood test results of four cases showed that: four people carried SARS virus IgG antibodies, of which two were discharged with higher antibody levels (patients 5 and 6) and two which were hospitalized had lower antibody levels (patients 3 and 4) (Wuhan, Chinese Academy of Sciences) Virology Institute)”. Xu's Master's thesis, Huang's Ph.D. thesis, and Ge et al. (10), all report the dominance of Chinese horseshoe bats (*Rhinolophus sinicus* and *Rhinolophus affinis*) in the mine. The Kunming Institute of Zoology also confirmed that the six patients were exposed to Chinese horseshoe bats (*Rhinolophus* species). *Rhinolophus* species harbor SARS-like coronaviruses (11). The blood biochemical analysis from the pneumonia patients indicated elevated markers such as Serum Amyloid A (SAA) with a normal range of PCT (procalcitonin), which suggested that the patients had a viral infection. The treatment given to the pneumonia patients included antivirals (ganciclovir, acyclovir injections), steroids (methylprednisolone), antibiotics (meropenem, vancomycin, etc.), antifungals (caspofungin, fluconazole), and anti-thrombotic medicines (warfarin, low molecular weight heparin). The thesis concludes that severe pneumonia in miners was due to SARS-like CoV from horseshoe bats. Dr. Nanshan's conclusion that the Mojiang miners pneumonia appeared to be primarily viral and that it was most probably due to bat-related coronaviruses, is noteworthy.

### Mojiang Mine and RaTG13

After the outbreak, WIV conducted longitudinal surveillance of the bat coronaviruses in the Mojiang mine (10). The mineshaft had six bat types of which the highest number of *Rhinolophus* sp. (horseshoe bats) were sampled. Sample collections were done four times between August 2012 and July 2013. A total of 150 alphacoronaviruses and only two betacoronaviruses, of which only one was SARS-like betacoronavirus (CoV/4991), were detected (10). The same virus 4991 was renamed as RaTG13, which is the next genetic relative of SARS-CoV-2 (12).

## Discussion

The retrospective analysis of the illness in the miners greatly resembles COVID-19 in the following aspects (Table 1B).

**Table 1B:** Common features observed in the six pneumonia patients and COVID-19.

**Features**	**COVID-19 (13, 14)**	**Six pneumonia patients (7) (master thesis 2013)**
**Major symptoms**		
Fever	✓	✓
Dyspnoea/Fatigue	✓	✓
Cough	✓	✓
**Minor symptoms**		
Sputum/bloody sputum	/in some cases	✓
headache	(in some)	(in some)
ARDS	✓	✓
**Laboratory results**		
lymphocytes	decrease	decrease
Serum amyloid A protein, mg/L	High values	High values
D-dimer, mg/L	High value	High value
**Radiology**		
Chest C. T. scan prominent picture	Ground glass opacities, bilateral pneumonia, peripheral consolidation	Ground glass opacities, bilateral pneumonia, peripheral consolidation
**Complications**		
Pulmonary thromboembolism	✓	✓
Vascular complications	✓	✓
Hypoxia	✓	✓
Secondary infections (bacterial, fungal)	✓	✓
Role of age	✓	✓
Co-morbidities	✓	✓
Male sex	✓	All were males
Reason of death	Cardiac arrest, ARDS, pulmonary failure	Cardiac arrest, ARDS, pulmonary failure

**1. The radiological picture** seen in the CT scans of COVID-19 patients (13) and miners cases (7) is very similar, which includes ground-glass opacities, peripheral consolidation, and clear indications of bilateral pneumonia (characteristic in COVID-19). This is highly evident on pages 25, 26 and 35, 37 in the translation (https://www.documentcloud.org/documents/6981198-Analysis-of-Six-Patients-With-Unknown-Viruses.html).

2. **Elevated D-dimer values and pulmonary thromboembolism**, a complication seen in COVID-19 were also found in three of the six miners in 2012 (7). The use of heparin, warfarin, and anticlotting drugs was successful in treating the respiratory condition in the fourth miner. Similarly, in COVID-19, pulmonary thromboembolism and blood clotting have been a serious complication.

3. **Lymphocytopenia**, that is, low lymphocyte counts are another common feature characteristic of a viral disease, and common in both the miners' pneumonia cases and COVID-19.

4**. The similarity in treatments:** Treatment given to the miners were antivirals, steroids, mechanical ventilation, antibiotics (for treating the secondary bacterial infections), and antifungals (for treating the secondary fungal infections). Anti-thrombotic agents like warfarin, heparin were also given in the case of patient 4 who successfully recovered. Very similar treatments are given for treating COVID-19 where an array of antivirals, steroids, blood thinners, antibiotics, and antifungals are given (in conjunction with the secondary infections).

**5. Elevated Serum Amyloid A protein**: is an inflammatory marker that shows characteristic high values in cases of viral infection. A high SAA value or an increasing trend is an indicator of bad prognosis in the case of COVID-19 (15). In the miners' pneumonia, this marker showed high initial values in the first four serious patients and later showed peaks of up to 1,000–1,200 mg/L in some cases.

(details of the similarities are given as Supplementary Informations B and C).

Based on the detailed evidence presented in the Master's thesis (7) and the Ph.D. thesis (9) and the discussion presented here, we do not think that fungus was the primary reason for the illness. Dr. Nanshan predicted the miners' illness to be a primary interstitial viral pneumonia (high probability) with invasive aspergillosis as a secondary infection (a condition commonly observed in COVID-19) (16). We think that if it was a fungal disease, only antifungals could have cured the illness. Vascular complications such as elevated D-dimer and thromboembolism are not common in fungal disease and have been observed in the miners' illness and COVID-19 (14). Elevated SAA (serum amyloid A) and declined lymphocytes are indicative of the fact that it was primary viral pneumonia (Supplementary Information C).

## Questions

As has been stated, the miners' samples were sent to WIV for SARS Ab testing (7, 9), the same institute that also conducted surveillance of the bat coronaviruses in the Mojiang mineshaft (10). The link between the SARS-like CoV (4991/RaTG13) from mine where lethal pneumonia cases occurred, has not yet been discussed in scientific papers by the WIV laboratory before February 2020. We are curious to know what kind of samples the WIV received from the Mojiang miners, along with other questions, such as whether the samples are still stored in WIV, and whether they are available for study by other researchers. It would also be of particular value to know whether any viruses were isolated and if there is any DNA/RNA available from these samples. It would also be useful to know if PCR was performed on the miners' samples and available sequences. According to Huang's Ph.D. thesis, four miners tested positive in an Ab test against SARS-like CoV (Supplementary Material). However, further questions remain as to which antigen was used for the Ab detection in the pneumonia patients and what was the exact protocol used. Why is this information not available in any of the seroprevalence studies by WIV? Why were the severe pneumonia cases in 2012 not mentioned in any of the WIV publications before 2020? Were any SARS-like CoV isolated from the bat fecal samples collected in 2012–13? Why were the Mojiang miners pneumonia cases in 2012 not reported to any public health agency like the WHO? Why did programs like PREDICT not mention the lethal pneumonia cases as a mini-outbreak? Was the mineshaft in Mojiang closed, when? According to the literature, three research groups went to the Mojiang mine to collect samples between 2012 and October 2014 (5, 9, 10). The mine was promptly closed as per the (2). Why was the Mojiang mine being visited by researchers until October 2014? Questions also remain as to why Dr. Shi attributed the outbreak in Mojiang to a fungus in the interview with Scientific American. Was the mine open for researchers and were any samples brought after 2014? Did any of the researchers who visited the Mojiang mineshaft get infected by any coronavirus between 2012 and 2019? Are there any whole genome sequences available for SARS-like CoV originating from this mine? Why is the pathogen database (http://www.viruses.nsdc.cn/chinavpi/) associated with the project (2013FY113500) (10) not accessible anymore?

## Conclusions

The striking similarities between the Mojiang pneumonia cases and COVID-19 are noteworthy, as is the fact that RaTG13/CoV4991, the next genomic relative of SARS-CoV-2 was found in the same mineshaft. The Master's thesis by Li Xu concludes that the pneumonia-illness in the miners was due to a SARS-like CoV from horseshoe bats. The remote consultation and diagnosis by a prominent pulmonologist in China, Dr. Nanshan, adds credibility to the diagnosis of the pneumonia cases in 2012. Although we cannot say that RaTG13 or SARS-CoV-2 infected the miners, there is a high chance that it could be a virus quite similar in genetic composition to these two. The coincidence between the 2012 illness in Mojiang miners, the subsequent samplings, and finding the nearest SARS-CoV-2 relative from this single mine warrants further inquiry, and the data along with the full history of this incident would be invaluable in the context of the current pandemic.

## Data Availability Statement

All datasets presented in this study are included in the article/Supplementary Material.

## Author Contributions

MR: conceptualization. MR and RB: Writing of the paper. Both authors reviewed and approved the final version.

## Conflict of Interest

The authors declare that the research was conducted in the absence of any commercial or financial relationships that could be construed as a potential conflict of interest.
