# Supplementary material for: Lethal Pneumonia Cases in Mojiang Miners (2012) and the Mineshaft Could Provide Important Clues to the Origin of SARS-CoV-2
Source: Front Public Health. 2020 Oct 20;8:581569. doi: 10.3389/fpubh.2020.581569 (PMC7606707; doi:10.3389/fpubh.2020.581569)
Supplement: Supplementary file 1 [file Table_1.DOCX]

**Supplementary Information A**

**Details of the lethal pneumonia illness in the six Mojiang miners**

1. The **first patient**, 63 years old, was exposed to the mine for 14 days. During admission to the hospital, he reported having signs of fever, cough, dyspnea, chest pain, and hiccups for more than 10 days. The illness progressively developed and he showed an increasing tendency of fever with a decline in lymphocytes. The condition worsened and the patient died after 12 days of admission. This patient was the oldest of all and had a tumor history (co-morbidity). The discharge (death) diagnosis was: severe pneumonia, sepsis, septic shock, abdominal infection, and cardiac arrest.

2. The **second patient**, aged 42 years, worked for 14 days in the mine. He was admitted to the hospital after cough and fever for ~15 days. He reported that he was also exposed to a lot of bat waste. The CT scans showed severe pneumonia in both the lungs. The condition worsened and after ~40 days the heart showed poor outline and indications of deep vein thrombosis. During the disease, the patient's lymphocytes decreased suggesting his immune function was impaired. He also had chronic Hepatitis B infection (co-morbidity). The arterial blood gas analysis suggested that he has persistent respiratory failure and poor oxygenation. The patient died after 48 days in the hospital and the diagnosis on discharge was respiratory and cardiac arrest, severe pneumonia, respiratory failure, sepsis, and death.

3. The **third patient** was 45 years old, male, and worked for 14 days in the mine. After 10 days he developed a cough, chest tightness, and shortness of breath with fever and sore limbs and headache. A remote video consultation by the famous doctor, Dr. Zhong Nanshan, the most expert doctor in China for SARS and Covid-19 was provided after ~52 days. Dr. Nanshan diagnosed the patient to have interstitial pneumonia (primarily of viral origin), a possibility of secondary infection (invasive pulmonary aspergillosis), and ordered to do a swab testing and SARS antibody testing (to be carried in Wuhan Institute of Virology). Also, he asked to confirm with the Kunming Institute of Zoology for confirmation of the type of the bat. He died after 109 days after admission to the hospital and treatment. In the case of patient 3, the discharge diagnosis after his death was: severe pneumonia; multiple organ failure; aspiration lung injury; acute respiratory distress syndrome (ARDS), interstitial pneumonia (highly viral probabilities); invasive pulmonary aspergillosis (secondary) followed by death.

**4. Patient 4**, aged 46 years worked for 14 days in the mine after which he developed a cough, sputum, and fever. In the CT scans, he showed bilateral interstitial pneumonia of severe kind. There were multiple ground glass exudates in both lungs, the lower lungs showed consolidation and a small number of pleural effusions on both sides. Similar to patient 3, this patient also received remote video consultation by Dr. Zhong Nanshan. He diagnosed him with interstitial pneumonia (viral possibility high) and invasive aspergillosis (secondary). The day after the antithrombotic treatment with warfarin, the patient's respiratory function was significantly improved, indicating that anticoagulation and antithrombotic treatment have an effect. Since the viral pneumonia was diagnosed and the patients had worked in the environment of bat feces, it was suggested to determine the bat types, pharyngeal dipstick or swab test, and SARS antibody test, by Dr. Nanshan. After struggling for 107 days as per the thesis actual for 137 days, and receiving a prolonged treatment he was discharged live.

**5 and 6. Patients 5 and 6**: These patients were relatively young (32/33 years) and exposed to the mine only for a short time (4/5 days). After admission, patients 5 and 6 were given antibiotics, anti-inflammatory, and anti-viral treatment. Patient 6 was not given antifungal drugs and still, the condition improved. Therefore, it was concluded that the initial onset of the disease was very unlikely to be caused by a fungal infection. Both of the patients were discharged after 24/26 days (Table 1A).
