# Supplementary material for: Lethal Pneumonia Cases in Mojiang Miners (2012) and the Mineshaft Could Provide Important Clues to the Origin of SARS-CoV-2
Source: Front Public Health. 2020 Oct 20;8:581569. doi: 10.3389/fpubh.2020.581569 (PMC7606707; doi:10.3389/fpubh.2020.581569)
Supplement: Supplementary file 2 [file Table_2.DOCX]

**Supplementary Information B**

**1. Clinical symptoms**

**Common symptoms seen in the Mojiang miners pneumonia:** Cough, fever, fatigue, sore limbs (in all except one), sputum, and severe pneumonia. In some patients, bloody sputum, headache, and chest pain were seen [1].

**Common symptoms seen in COVID-19:** The most common symptoms of COVID-19 are the same: fever, cough, and tiredness. Other symptoms that are less common and may affect some patients include aches and pains, nasal congestion, diarrhea, headache, conjunctivitis, etc. (as per the WHO).

2. **Age and co-morbidities** increased the complications and resulted in death, in both the Mojiang miners pneumonia cases (though the patient number is small) and COVID-19.

**3. The radiological picture** seen in the CT scans of COVID-19 patients [2] and miners cases [1] is very similar which includes ground-glass opacities, peripheral consolidation, and clear indications of bilateral pneumonia.

4. **Blood clotting and pulmonary thromboembolism**, a complication seen in COVID-19 were also found in three of the six miners in 2012 [1]. The use of heparin, warfarin, and anticlotting drugs was successful in treating the respiratory condition in the 4^th^ miner. In the case of COVID-19, pulmonary thromboembolism and blood clotting have been a serious complication. Anti-thrombotic medicines or blood thinners are given in COVID-19 to treat thrombotic complications such as pulmonary thromboembolism.

5. **Lymphocytopenia**, i.e. low lymphocyte counts is another common feature in both, the miners pneumonia cases and Covid-19. Especially the T cell depletion is a common feature observed in both the diseases.

6**. The similarity in treatments:** Treatment given to the miners were: antivirals, steroids, mechanical ventilation, antibiotics (for treating the secondary bacterial infections) and antifungals (for treating the secondary fungal infections). Also, anti-thrombotic agents like warfarin, heparin were given in case of patient 4 who successfully recovered. Very similar treatment is given for COVID-19 where an array of antivirals, steroids, blood thinners, antibiotics, and antifungals are given (in conjunction with the secondary infections).

**7. Secondary infections**: In the case of the miners pneumonia illness, most of the patients had secondary infections due to bacteria (*Acinetobacter baumannii, Klebsiella pneumoniae*) or secondary aspergillosis [1]. This scenario is very similar to COVID-19 where secondary bacterial infections due to similar organisms [3] and aspergillosis infections are commonly being observed [4].

8. **Elevated SAA values**: The Mojiang miners pneumonia cases showed that the serum amyloid A protein (SAA) was elevated in the case of the first 4 patients and showed very high values (~198-400 mg/L) just after admission. These values are exceptionally high and show poor prognosis if considered to be similar to COVID-19 [5]. The SAA protein is characteristically elevated in the case of COVID-19 patients showing poor prognosis and is used as a marker for the declining condition [5].

9. **Different nature to SARS1**: In the case of SARS1, mostly unilateral pneumonia is seen in the CT scans [6]. In contrast to that, both pneumonia illness in miners and COVID-19 show mostly bilateral pneumonia. The kind of complications seen in the pneumonia of miners, e.g. pulmonary thromboembolism, are seen both in pneumonia illness in miners and COVID-19. Also, Dr. Nanshan did not classify the pneumonia cases as SARS1. (We have not compared the symptoms with MERS, as MERS emerged in September 2012 in Saudi Arabia, later than the miners pneumonia).

[1] L. Xu, 'The analysis of 6 patients with severe pneumonia caused by unknown viruses' The First Clinical Medical College of Kunming Medical University, Kunming Medical University, China, China, 2013 Master's Thesis, pp. 66.

[2] H. Shi, X. Han, N. Jiang, Y. Cao, O. Alwalid, J. Gu, Y. Fan, and C. Zheng, Radiological findings from 81 patients with COVID-19 pneumonia in Wuhan, China: a descriptive study. The Lancet 20 (2020) 425-434.

[3] C.-C. Lai, C.-Y. Wang, and P.-R. Hsueh, Co-infections among patients with COVID-19: The need for combination therapy withnon-anti-SARS-CoV-2 agents? J. Microbiol. Immunol. Infect. 53 (2020) 505-512.

[4] S. Johns, COVID-19 patients could be at greater risk of fungal infections, researchers say, Imperical College London, London, UK, 2020.

[5] H. Li, X. Xiang, H. Ren, L. Xu, L. Zhao, and e. al, Serum Amyloid A is a biomarker of severe Coronavirus Disease and poor prognosis. J. Infect. 80 (2020) 646-655.

[6] M. Hosseiny, S. Kooraki, A. Gholamrezanezhad, S. Reddy, L. Myers, H. M, and Kooraki S, Radiology Perspective of Coronavirus Disease 2019 (COVID-19): Lessons From Severe Acute Respiratory Syndrome and Middle East Respiratory Syndrome. American Roentgen Ray 214 (2020) 1078-1082.
