# Supplementary material for: Lethal Pneumonia Cases in Mojiang Miners (2012) and the Mineshaft Could Provide Important Clues to the Origin of SARS-CoV-2
Source: Front Public Health. 2020 Oct 20;8:581569. doi: 10.3389/fpubh.2020.581569 (PMC7606707; doi:10.3389/fpubh.2020.581569)
Supplement: Supplementary file 3 [file Table_3.DOCX]

**Supplementary Information C**

**Elevated biomarkers in the miners’ illness correlated with viral disease and/or also specifically with COVID-19**

**1. High Serum Amyloid A (SAA) (inflammation marker)** elevated in viral disease, with moderate CRP. High CRP is correlated to bacterial disease.

Information about SAA:

a. SAA belongs to the apolipoprotein family, mainly from the liver, plays an important role in inflammatory response and lipid metabolism, and is one of the main acute phase proteins of the body.

b. The difference between SAA and CRP is that in viral infectious diseases, SAA is significantly increased, but CRP is not elevated; in bacterial infectious diseases.

**SAA levels in the miners pneumonia cases:**

In the case of these six patients, it was found that the SAA of the first four critical patients during admission were exceptionally high: 198, 398, 434 and 380 mg/L, respectively.

SAA of these four patients increased in some cases upto 1000 or 1200 mg/L in case of patients 3 and 4.

All these values were very high during admission, indicating that they already were suffering from viral illness. In case of the miners, all the three miners who died showed a higher value of SAA even after the treatment suggesting that the disease progressed to more severe and critical form. The two less ill patients showed relatively better SAA values (44 and 79 mg/L) initially indicating that their disease was moderate.

SAA ranges between 123-181 mg/l between mild, moderate and severe cases (COVID-19).

Normal SAA value is under: 10mg/L

**2. Elevated D-dimers and pulmonary embolism**

Information:

D-dimer is commonly elevated in patients with COVID-19. D-dimer levels correlate with disease severity and are a reliable prognostic marker for in-hospital mortality in patients admitted for COVID-19 (Yao et al 2020).

.

**D-Dimers in the miners pneumonia cases**: Elevated D-dimers and indications of pulmonary embolism was seen in case of patients 1, 2 and 4.

D-dimers were significantly high in miners and were measured in 3-4 patients who showed indications of pulmonary embolism or clotting.

1. Patient 1 admission to death: **7.2, 3.6, 7.0, 5.0** µg/ ml= mg/L (result: death)

2. Patient 4 admission to death: **8.9, 6.9, 3.7, 3** µg/ ml (measured till August (discharged early sept). This patient was given anti-coagulant (low molecular weight heparin).

3, Patient 6 **3.5** µg/ ml (measured in middle) discharged after recovery

Normal D-dimers value is: 0.5 µg/ ml= mg/L

**Radiological features correlated with COVID-19 and miners pneumonia**

**CT Scans:**

**CT scan with ground glass opacities and peripheral consolidation were also characteristically observed. The involvement of lower lobes is also characteristic in COVID-19 and was observed in the miners.**

One of the main references:

# <https://medicalxpress.com/news/2020-03-chinese-chest-ct-coronavirus-disease.html> Chinese researchers detail chest CT findings in coronavirus disease (COVID-19) pneumonia

Ground-glass opacities (GGO) (86.1%) or mixed GGO and consolidation (64.4%) and vascular enlargement in the lesion (71.3%), peripheral distribution (87.1%) and bilateral involvement (82.2%) and be lower lung predominant (54.5%) and multifocal (54.5%) are the main features in COVID-19.

In case of miners, the most severe patients (3 and 4) showed characteristic features of ground glass opacities, peripheral consolidation and bilateral pneumonia, and showed the typical CT pictures seen in COVID-19 patients.

Page 24, 25, 26 –Patient 3

Pages 35-40 Patient 4

These pictures were shown to a radiologist and he confirmed that these were very similar to that of the COVID-19 patients.
